# Integrative Medicine for Patients with Diabetes and Mental Health Disorders: A Narrative Review

**DOI:** 10.1007/s11920-025-01623-7

**Published:** 2025-07-14

**Authors:** Sameer Kassem, Noah Samuels, Eran Ben-Arye

**Affiliations:** 1 Rappaport Faculty of Medicine, Technion-Israel Institute of Technology, Haifa, Israel; 2 Department of Internal Medicine, Carmel Medical Center, Clalit Health Services, Michal 7, Haifa, Israel; 3 Center for Integrative Complementary Medicine, Faculty of Medicine, Shaare Zedek Medical Center, Hebrew University of Jerusalem, Jerusalem, Israel; 4 Integrative Oncology Program, The Oncology Service, Lin, Carmel & Zebulun Medical Centers, Clalit Health Services, Haifa, Israel

**Keywords:** Mental health, Diabetes, Integrative medicine, Complementary medicine, traditional Chinese medicine, Mind-body

## Abstract

**Purpose of Review:**

We review the literature on the interconnections between diabetes and mental health disorders, such as depression and anxiety, highlighting recent findings and potential integrative medicine (IM) interventions for improving patient care in individuals with both comorbidities.

**Recent Findings:**

Underscore the bidirectional relationship between diabetes and depression, with each condition exacerbating the other with associated poorer clinical outcomes. Pathophysiological mechanisms like hypothalamic-pituitary-adrenal (HPA) axis dysregulation and chronic inflammation contribute significantly. IM approaches, including mind-body therapies, dietary modifications, and herbal medicine, show promise in enhancing both glycemic control and mental health well-being.

**Summary:**

IM offers a holistic, patient-centered approach potentially improving care for individuals with diabetes and mental health disorders. While evidence supports the efficacy of various integrative therapies, further rigorous research is needed to standardize these interventions and ensure their effective integration into conventional medical practice.

## Introduction

The prevalence of diabetes has continued to increase over recent decades, with an estimated 828 million adults worldwide diagnosed with this condition in 2022 [1]. The implications of the diabetes pandemic on the risk for developing mental health disorders such as depression and anxiety are significant. According to the International Diabetes Management Practices Study, depressive symptoms were reported in 30.7% of patients with type 1 and 29.0 to 46.7% of patients with type 2 diabetes in developing countries. Depending on the treatment regimen, the prevalence of moderate-to-severe depression among patients with diabetes can reach as high as 11.2% [2]. A meta-analysis of observational studies estimated that approximately 28% of patients with type 2 diabetes experience depressive symptoms, with the risk increasing among younger patients and those from Asia and Australia [3]. In the United States, data from the 2019 Behavioral Risk Factor Surveillance System found a significantly higher incidence of depression among adults with diabetes (29.2%) than among those without diabetes (17.9%) [4]. Similar findings have been reported in other regions such as the United Kingdom, where depression rates in patients with type 2 diabetes increased from 29% in 2006 to 43% in 2017 [5]. In the INTERPRET-DD study, which included participants from 14 countries, it was shown that 10.6% of patients with type 2 diabetes had been diagnosed with a major depressive disorder, with 17.0% reporting moderate-to-severe depressive symptoms [6]. Medical parameters related to the severity of diabetes were found to be associated with a significant risk for depression, specifically insulin use and the presence of cardiovascular disease, for which the correlation was most significant [7].

The 2025 American Diabetes Association (ADA) guideline estimates that depressive symptoms and disorders affect one in four people with type 1 or type 2 diabetes, recommending routine screening for depressive symptoms in all patients with diabetes. Screening should be conducted at least once yearly, more frequently for patients with a history of depression or significant changes in medical status. The ADA guideline emphasizes that depression can significantly impact diabetes management and overall quality of life (QoL), requiring a multi-disciplinary integrated approach to care which includes behavioral healthcare professionals, in collaboration with the diabetes treatment team [8].

The association between diabetes and mental health may, in part, be explained by a wide range of pathophysiological processes which have been associated with depression, and which have been shown to increase the risk for developing diabetes. An example of this is chronic and persistent psychological stress, which is associated with dysregulation of the hypothalamic-pituitary-adrenal (HPA) axis resulting in elevated cortisol levels that promote insulin resistance and hyperglycemia [9]. Chronic inflammation has also been seen as a potential cause for the development of diabetes, with depression associated with increased levels of pro-inflammatory cytokines such as IL-6 and TNF-α. These cytokines can impair insulin signaling pathways, contributing to insulin resistance and the subsequent development of type 2 diabetes [10, 11]. Levels of neurotransmitters such as serotonin, dopamine and norepinephrine are altered in patients with depression, impairing neurotransmission and affecting glucose metabolism and insulin sensitivity [12].

Other behavioral factors associated with depression, such as poor diet, physical inactivity, and non-adherence to medical treatment, may further increase the risk for weight gain and the development of metabolic syndrome, both considered significant risk factors for type 2 diabetes [13]. Depression can also lead to changes in gut microbiota composition, which in turn can affect metabolic health and increase the risk for diabetes through mechanisms related to short-chain fatty acid and tryptophan metabolizing pathways [14].

In summary, there are many pathophysiological mechanisms associated with depression which are also known to promote the development of diabetes. These include HPA axis dysregulation, inflammation, neurotransmitter imbalances, adverse health behaviors, and microbiome alterations. The large number of interconnected pathways highlights the complexity of the relationship between diabetes and disorders of mental health.

### The Burden of Diabetes and Mental Health Co-Morbidity

Epidemiological studies have consistently demonstrated a bi-directional relationship between diabetes and depression in which each condition exacerbates the other, leading to poorer clinical outcomes. Golden et al. showed that depressive symptoms are associated with an increased risk for developing type 2 diabetes; and that type 2 diabetes is associated with an increased risk for developing depressive symptoms [15]. Similarly, Chen et al. found that individuals with diabetes had a higher incidence of depression, and that those with depression had a higher incidence of diabetes, reinforcing the bi-directional relationship between these two conditions [16].

The economic burden of a co-diagnosis of diabetes and depression can be substantial, with numerous studies indicating increased healthcare costs and resource utilization in patients with both disorders. In the United States, Egede et al. found that patients with both diabetes and depression incur significantly higher health-related costs when compared to those with either condition on its own, exceeding the sum of expenses for each diagnosis separately [17]. In a second study by Egede et al., patients with diabetes and depression were shown to require healthcare expenditures which were 4.5 times higher than those incurred among patients with diabetes alone. This significant increase in cost has been attributed to a higher rate of ambulatory care utilization, as well as the increased need for prescription drugs [18]. In Germany, Brüne et al. reported that patients with both diabetes and depression face significantly higher annual healthcare costs when compared to those without either of these conditions. The increase in expenditure was attributed primarily to accompanying co-morbidities, rather than the costs of mental healthcare, further increasing the overall economic burden [19].

The intersection between diabetes and depression/anxiety presents a number of challenges to the healthcare professional, with unmet patients’ needs [20]. These include psychosocial-related issues, such as increased stress and social isolation due to the stigma associated with both diabetes and mental health-related disorders. This can make it even more difficult for the patient to seek out the necessary help in a non-judgmental setting. In a study by Kelly et al. among predominantly type-1 diabetes patients, 86% self-reported a sense of anxiety and/or depression. The overwhelming majority of respondents stated that the best thing their diabetes healthcare providers could do to help would be to simply ask them about their mental well-being; listen with empathy and without judgement; and implement skills which would help them better understand psychosocial issues common among patients with diabetes [21]. A lack of integrated care and poor communication between healthcare providers and patients with diabetes has often led to a less-than-effective setting of patient support. Tanenbaum et al. reported that patients with type 2 diabetes describe multiple sources of distress, including a lack of support and understanding from others; difficulty in communicating with their healthcare providers; and distress from the need to make lifestyle changes [22].

Diabetes research has shown that actively engaging in patient care is essential, though this may be challenging due to a number of factors which include set beliefs about conventional medical treatments for this condition; a high level of distress associated with diabetes; low self-efficacy and self-management for treating the patient’s diabetes, all of which are associated with less effective glycemic control [23]. It is believed that through the integration of complementary medicine practices with conventional medical care, healthcare providers can deliver a more personalized and comprehensive treatment program, creating a synergy which can more effectively improve the patient’s overall health and quality of life, especially for those faced with both diabetes and mental health-related issues.

### Integrative Medicine for Diabetes and Mental Health

A 2002 study by Egde et al. showed that U.S. patients with diabetes were 1.6 times more likely to use complementary and alternative medicine (CAM) than those without diabetes [24]. In developing countries, the use of CAM and traditional medicine is widespread, with up to 62% of patients with diabetes reporting using at least one type of CAM, usually in a complementary rather than alternative setting [25]. In Saudi-Arabia, 26% reported CAM use in the context of their diabetes care, with mind-body modalities the most prevalent modality being used [26]. In order to explore the feasibility of an integrative medicine (IM) model in primary care, Ben-Arye et al. found that CAM use among 485 patients with diabetes was significantly more prevalent than among those without diabetes, more so among patients who reported being more religious in their beliefs [27].

There is still a need for more extensive research examining the inter-relationship between diabetes, CAM use and mental-spiritual care. In 2004, Kligler suggested an integrative model for an optimal healing environment in the care of patients with diabetes which includes biophysical, behavioral, psychological, social and spiritual components [28]. Two decades later, the Chair of the Board of Directors for the Academic Consortium Integrative Medicine and Health, Amy Locke, described integrative medicine as patient-centered paradigm that “reaffirms the importance of the relationship between practitioner and patient, focuses on the whole person, is informed by evidence, and makes use of all appropriate therapeutic and lifestyle approaches, healthcare professionals and disciplines to achieve optimal health and healing” [29]. Whether the IM model of care is feasible in real-life diabetes care needs to be further investigated, especially for patients diagnosed with both diabetes and mental illness. The following subsections review key IM modalities to be considered within a comprehensive model of care for patients with a co-morbidity of diabetes and mental health-related disorder.

#### Mind-Body Interventions

Mind-body therapies include a wide range of interventions which are rooted in both Western and Far-Eastern medical practices. They include modalities such as breathing and relaxation techniques; guided imagery; meditation and mindfulness; and others. Mind-body therapies have been shown to enhance diabetes-related psychological and cardiometabolic outcomes, promoting self-management behavior [30]. In a systematic review and meta-analysis, Cooper et al. found that the use of modalities such as behavioral activation and cognitive behavioral therapy (CBT) was associated with a significant reduction in both glycated hemoglobin (HbA1c) levels and depressive symptoms [31]. In light of the extensive research on mind-body medicine, the ADA has recommended the integration of psychological interventions such as CBT, mindfulness-based approaches, and collaborative care for depressive symptoms and glycemic control in patients with diabetes [8].

Mindfulness-based stress reduction (MBSR) and mindfulness meditation have both been shown to significantly improve glycemic control and reduce depression, anxiety, and stress-related scores in patients with type 2 diabetes [32]. Stress reduction and improved mood are especially important for patients with diabetes, since both negatively impact diabetes self-management and glycemic control. In a study by Wang et al., an MBSR program significantly reduced the perceived levels of stress and anxiety, which in turn improved diabetes self-efficacy and self-management behavior [33]. Another study by Armani Kian et al. found that MBSR led to a significant reduction in fasting blood sugar and HbA1c levels, indicating better glycemic control [34]. A meta-analysis by Ni et al. concluded that MBSR and mindfulness-based cognitive therapy (MBCT) significantly improved mental health composite scores for overall QoL, depression, and HbA1c levels in people with diabetes [35]. A more recent meta-analysis by Fisher et al. found that MBSR effectively reduced anxiety and depression in patients with diabetes, though no significant improvement was observed with respect to HbA1c levels [36]. In a pilot study, Guo et al. observed that a hospital-based nurse-directed MBSR therapy for patients with diabetes significantly reduced distress and HbA1c levels [37].

The mechanism for the effects of mind-body interventions on glucose levels may be explained through modulation of the hypothalamic-pituitary-adrenal (HPA) axis, with stress reduction leading to reduced cortisol levels and thus decreased insulin resistance. MBSR and mindfulness meditation have been shown to enhance the HPA axis habituation to stress, which is considered central to the reduction of the chronic stress response [38]. Wetherell et al. found that MBSR significantly reduced cortisol levels in older adults with high baseline cortisol levels [39], most likely through modulation of the HPA axis.

Reduction of serum cortisol levels resulting from mind-body interventions such as MBSR is especially important in patients with type 2 diabetes, for whom chronic hypercortisolemia is a major factor leading to increased insulin resistance. Obaya et al. showed that the combination of mindfulness meditation with aerobic exercise significantly reduced both fasting blood glucose and cortisol levels in women with type 2 diabetes, suggesting improved insulin sensitivity [40]. Aguilar-Raab et al. observed that mindfulness interventions can reduce both perceived stress from daily life and cortisol levels, supporting the role of mindfulness in attenuating the physiological stress response [41]. In a randomized controlled clinical trial (RCT) by Friis et al., an 8-week mindful self-compassion (MSC) program significantly reduced depressive symptoms and diabetes-related distress, leading to a decrease in HbA1c levels by nearly 1% [42].

Other mind-body modalities which should be considered for the treatment of patients with diabetes include QiGong and Tai Chi exercises, which originate from traditional Chinese medicine (TCM) and entail mindful movement and breathing. In a systematic review with meta-analysis of studies on patients with type 2 diabetes, both Tai Chi and QiGong were associated with decreased HbA1c levels and improved QoL, with the latter also leading to significant relief of depression [43]. In a study by Putiri et al., Yi Ren Medical Qigong was found to reduce perceived stress and possibly relieve depression in patients with type 2 diabetes [44].

#### Yoga and Physical Activity

Yoga is a unique IM modality which is rooted in traditional Indian Ayurvedic medicine, combining elements of mind-body, movement, and relaxed breathing. Yoga practice includes physical postures (Asana), breathing techniques (Pranayama), and meditation (Dhyana). In the U.S. Healthy Active and in Control (HA1C) feasibility study, Bock et al. found that Yoga significantly reduced diabetes-related emotional distress and improved QoL when compared to standard exercise [45]. The study also found that patients practicing Yoga experienced a greater improvement in mindfulness, with reduced psychological distress.

The impact of Yoga on regulation of the autonomic nervous system can be seen through its effects on objective heart rate variability (HRV) measurements [46]. Danasegaran et al. found that a 12 week Yoga intervention for male patients with type 2 diabetes led to significantly improved HRV measurements with reduced cardio-metabolic risk, including markers of myocardial stress and inflammation [47]. The HRV measurements observed reflect better autonomic control, which is seen as crucial for managing stress and improving emotional well-being. In their systematic review, Ruiz-Ariza et al. addressed the effectiveness of mind-body training (including Yoga) in reducing anxiety, depression, and stress levels in adults with type 2 diabetes [48]. Yoga practices have also been associated with the reductions of stress hormones such as cortisol. A systematic review by Pascoe et al. found that Yoga lead to better regulation of the HPA axis, resulting in decreased cortisol levels and improved mood [49].

#### Acupuncture and Acupressure

Acupuncture and acupressure are manual TCM modalities entailing the insertion of acupuncture needles into or applying localized pressure at specific points along energy (Qi) channels. Acupuncture treatment has been shown to induce a wide range of physiological processes, include increased expression of brain-derived neurotrophic factor (BDNF), which plays a vital role in both diabetes and depression. A review by Zhang et al. examining co-morbidity with diabetes and depression concluded that acupuncture may exert its effects via the BDNF-TrkB-ERK-CREB signaling pathway, improving symptoms and glycemic control [50]. In a study by Cheok et al., acupuncture was found to further reduce insulin resistance when added to conventional treatment, as measured by HOMA-IR and health-related quality of life (HRQoL) in patients with type 2 diabetes [51].

In addition to the above mechanisms, acupuncture has also been shown to modulate the levels of inflammatory cytokines and neurotransmitters. In a model of chronic stress in rats, Lu et al. showed that that acupuncture significantly decreased the pro-inflammatory cytokines IL-1β, IL-6, and TNF-α in the hippocampus and prefrontal cortex, both associated with depressive symptoms [52]. In a mouse model of depression, Jung et al. found that acupuncture improved depression-like behavior and modulated lipid metabolism and inflammatory interactions, including leptin signaling [53].

A systematic review and meta-analysis by Liu et al. included 36 studies with a total cohort of 2,739 patients with painful diabetic neuropathy. The review concluded that acupuncture significantly reduced pain intensity and depression scores, with improved QoL [54]. In an RCT from Egypt, El-Shamy et al. found improved gestational diabetes outcomes following acupressure at 12 weeks (75 g oral glucose tolerance test, OGTT; insulin resistance; insulin dosing), though the study did not examine the effect of the intervention on mental health-related concerns [55]. However, in two studies conducted in Iran, acupressure was shown to provide relief for anxiety in pregnant women with diabetes [56] and patients with type 2 diabetes, with improved fasting blood glucose and mean stress scores [57]. Finally in a study by Wei-Bo et al., a 12-week intervention with acupressure self-administered by patients with diabetes was shown to significantly reduce HbA1c levels and improve scores on the Diabetes-Specific Quality of Life Scale (DSQLS), which addresses mental health-related concerns, including depressed mood and anxiety about the future [58, 59].

#### Dietary Modifications and Nutritional Supplements

Dietary-based IM approaches in diabetes care may address both what the patient is eating (and what they need to avoid) as well as the context in which they are eating, taking into consideration other lifestyle and cultural aspects. In this regard, mindful eating may be seen within the context of a diabetes self-management educational intervention. According to Miller, “mindful eating helps individuals cultivate awareness of both internal and external triggers to eating, interrupt automatic eating, and eat in response to the natural physiological cues of hunger and satiety“ [60]. In a cross-sectional study in Turkey, mindful eating was associated with reduced body mass index (BMI) and risk for developing type 2 diabetes in young adults [61]. A similar effect was also shown in patients diagnosed with diabetes for at least six months [62].

In an RCT exploring the effect of a mindful eating intervention, compared with a diabetes self-management intervention, Miller et al. found a significant improvement in depressive symptoms in both groups [63]. Nutritional interventions, such as adopting a Mediterranean or plant-based diet, have been shown to help reduce systemic inflammation and improve both metabolic and mental health outcomes in patients with diabetes and depression [64]. These diets are rich in omega-3 fatty acids, antioxidants and fiber, all of which play an important role in modulating inflammation and supporting overall health. Omega-3 fatty acids, found in fatty fish and nuts, have been associated with reduced levels of pro-inflammatory cytokines such as IL-6 and TNF-α. The reduction in inflammation can improve insulin sensitivity and glycemic control, as well as alleviate depressive symptoms [64, 65].

Other antioxidants which are abundant in fruits, vegetables, and olive oil combat oxidative stress, another key factor in the pathogenesis of both diabetes and depression. By reducing oxidative stress, antioxidants help improve metabolic health and support neuroprotection, enhancing mental health outcomes [66, 67]. At the same time, dietary fiber such as that found in whole grains, legumes, and vegetables, supports gut health by promoting healthy gut microbiota. A balanced gut microbiota is also essential for reducing systemic inflammation and improving metabolic and mental health. Fiber intake is associated with lower levels of the inflammatory serum marker C-reactive protein (CRP) and improved insulin sensitivity, which in turn can mitigate the chronic inflammatory state associated with diabetes and depression [68, 69].

#### Herbal Medicine

The use of herbal medicinal compounds is a mainstay of traditional medicine, with extensive ethno-botanical research supporting their use in diabetes care [70]. Many herbal products such as St. John’s Wort (*Hypericum perforatum)* for depression, and cinnamon, nigella (black cumin), fenugreek, or berberine for glycemic control, have been shown to be effective. However, further research is still needed, and herbal compounds are not without potentially harmful effects, including herb-drug interactions which may compromise conventional medical care. For example, while St. John’s Wort has been shown to be as effective as second-generation anti-depressants in the treatment of mild-to-moderate depression [71], its role in diabetes is currently limited to pre-clinical research [72]. At the same time, the herb has been shown to significantly reduce serum levels of conventional drugs due to its effects on drug metabolism and absorption [72].

In addition to its beneficial effects on glycemic control, cinnamon has also been shown to improve cognitive function and increase brain insulin sensitivity in animal models, with implications for mental health benefits as well [73]. In a study taking place in a primary care setting, the daily addition of 1 gr cinnamon for 3 months was shown to significantly decrease HbA1c when compared with usual care alone [74]. A similar anti-diabetic effect of cinnamon was observed in an RCT conducted in Iraq among patients with poorly controlled type-2 diabetes, though the study did not examine mental health-related outcomes [75].

Clinical research has shown the herb Nigella (*Nigella sativa*) to have both antidepressant and anxiolytic effects [76]. Rahmani et al. examined the effect of the herb on depression and QoL in patients with diabetes undergoing hemodialysis, with possible beneficial effects on mental health-related outcomes [77].

The herb Fenugreek (*Trigonella foenum-graecum*) contains trigonelline, which has been shown in pre-clinical research to have neuroprotective, antidepressant, and anxiolytic properties [78]. In a placebo-controlled RCT conducted in India, a fenugreek seed extract was reported to significantly decrease fasting and postprandial glucose and HbA1c levels [79]. Another RCT from India examining healthy women with perimenopausal symptoms found an association between fenugreek supplementation and decreased rates of depression [80], with an indication for a similar effect on mental health-related disorders in diabetic patients as well [81].

Ashwagandha (*Withania somnifera*) is one of the most prominent herbs used in traditional Indian Ayurvedic medicine, and has been shown to reduce HbA1c levels in patients with diabetes [82]. A large body of research has shown this herb to be effective in the treatment of insomnia, anxiety, memory and cognitive decline and obsessive-compulsive disorder. Still, further research is needed in order to better understand the role of this herb for mental health-related concerns among patients with diabetes [83].

Finally, berberine is a compound found in hundreds of species from the *Berberis* family, and has been shown to alleviate depressive symptoms by modulating neurotransmitter levels, promoting hippocampal neurogenesis, and reducing inflammation [84]. Studies have demonstrated its efficacy in improving cognitive function and reducing depression in a mouse model of diabetes [85].

While the findings of the research examining the effects of herbal compounds on mental health and diabetes are extensive, there is still a need for further research to better understand their role in the treatment of patients with both medical conditions. Patients with diabetes frequently have other co-morbidities which need to be addressed as well, and the potential for negative herb-drug interactions requires special attention before their use can become standard practice.

### Clinical Implications

Integrative medicine provides a patient-centered, holistic approach which is tailored to the individual physical and mental needs of the patient. The implementation of these practices in the conventional clinical setting may enhance patient adherence to lifestyle modifications; improve both metabolic and mental health-related outcomes; reduce reliance on pharmacotherapy, thereby reducing adverse effects and drug interactions; and foster a collaborative care environment that addresses the multifaceted needs of patients with diabetes and comorbid mental health disorders.

However, there still remain a large number of challenges regarding standardization of IM interventions, provider training, and insurance coverage. While current evidence is promising, more rigorous RCTs are needed to firmly establish the efficacy and long-term benefits of IM approaches in the treatment of mental health-related disorders among patients with diabetes.

### Future Directions

Future directions for research on the treatment of mental health-related disorders in patients with diabetes should focus on the following approaches:

- Randomized clinical trials assessing the long-term impact of integrative interventions on glycemic control and mental health.
- Studies examining the cost-effectiveness of IM treatment programs to justify their incorporation into standard medical care.
- Investigations into the molecular and neurobiological mechanisms of the beneficial effects of IM interventions, with a potential for creating personalized integrative treatment protocols.
- Pragmatic, “real life” research of the patient-tailored and multi-modal setting of IM care, addressing the patient’s health-belief model, expectations and unmet QoL-related needs; and focusing on mental health-related concerns such as depression, anxiety, shame, self-blame and anger, among others.

## Conclusion

The IM setting, in which complementary therapies are provided as part of conventional diabetes care, can significantly help healthcare professionals address many of the challenges facing their patients suffering from both metabolic dysregulation and mental health-related disorders. The large body of evidence supporting this integration includes modalities such as mindfulness (including mindful eating), yoga, acupuncture, acupressure, nutritional modifications and herbal supplementations, which have been shown to positively impact both glycemic control and psychological well-being. As the field of IM continues to evolve, its role in managing chronic conditions such as diabetes and its complex interplay with mental health will likely become clearer, leading to the inclusion of these therapies in standard medical care for patients with both diagnoses.

## Data Availability

No datasets were generated or analysed during the current study.

## Key References

- Aschner P, Gagliardino JJ, Ilkova H, Lavalle F, Ramachandran A, Mbanya JC, et al. High prevalence of depressive symptoms in patients with type 1 and type 2 diabetes in developing countries: results from the international diabetes management practices study. Diabetes Care. 2021 May 7;44(5):1100–7. **This multinational cross-sectional real-world study reports depressive symptoms in 30.7% of patients with type 1 and 29.0 to 46.7% of patients with type 2 diabetes in developing countries. Depending on the treatment regimen**,** the prevalence of moderate-to-severe depression among patients with diabetes can reach as high as 11.2%.**
- American Diabetes Association Professional Practice Committee. 5. Facilitating Positive Health Behaviors and Well-being to Improve Health Outcomes: Standards of Care in Diabetes-2025. Diabetes Care. 2025 Jan 1;48(Supplement_1):S86–127. **The 2025 ADA guidelines estimate that one in four people with diabetes experience depression**,** recommending routine screening for all patients. They highlight that depression significantly impacts diabetes management and quality of life**,** necessitating a multidisciplinary**,** integrated approach.**
- Khawagi WY, Al-Kuraishy HM, Hussein NR, Al-Gareeb AI, Atef E, Elhussieny O, et al. Depression and type 2 diabetes: A causal relationship and mechanistic pathway. Diabetes Obes Metab. 2024 Aug;26(8):3031–44. **This review discusses possible mechanistic pathways linking depression and type 2 diabetes including altered levels of various neurotransmitters.**
- Brüne M, Linnenkamp U, Andrich S, Jaffan-Kolb L, Claessen H, Dintsios C-M, et al. Health Care Use and Costs in Individuals With Diabetes With and Without Comorbid Depression in Germany: Results of the Cross-sectional DiaDec Study. Diabetes Care. 2021 Feb;44(2):407–15. **This cross-sectional study reported that patients with both diabetes and depression face significantly higher annual healthcare costs when compared to those without either of these conditions.**
- Trief PM, Wen H, Burke B, Uschner D, Anderson BJ, Liu X, et al. Psychosocial Factors and Glycemic Control in Young Adults With Youth-Onset Type 2 Diabetes. JAMA Netw Open. 2024 Apr 1;7(4):e245620. **In this cohort study of young adults with type 2 diabetes**,** beliefs about medicines**,** high diabetes distress**,** and low self-management support were associated with high HbA1c**.
- Blanchette JE, Paquin F, Dobbs BN, Kiely RL, Hatipoglu B. Incorporating complementary therapies into diabetes care. J Clin Endocrinol Metab. 2025 Feb 25;110(Supplement_2):S137–46. **This recently published minireview reports that complementary therapies support diabetes-related psychological and cardiometabolic outcomes.**
- Liu J, Lin Y, Huang Y, Yang Q, Li X, Ye Y, et al. Efficacy and safety of acupuncture for painful diabetic neuropathy: a systematic review and meta-analysis. Front Neurol. 2024 Jun 5;15:1402458. **A systematic review and meta-analysis in patients with painful diabetic neuropathy concluded that acupuncture significantly reduced pain intensity and depression scores.**
